# Recent Advances and Challenges in 2D Metal-Free Electrocatalysts for N_2_ Fixation

**DOI:** 10.3389/fchem.2020.00437

**Published:** 2020-06-10

**Authors:** Xinyuan Xia, Bin Li, Shanshan Liu, Bo Tang

**Affiliations:** ^1^Key Laboratory of Molecular and Nano Probes, Ministry of Education, College of Chemistry, Chemical Engineering and Materials Science, Collaborative Innovation Center of Functionalized Probes for Chemical Imaging in Universities of Shandong, Institute of Molecular and Nano Science, Shandong Normal University, Jinan, China; ^2^College of Chemical Engineering and Safety, Binzhou University, Binzhou, China

**Keywords:** two-dimensional, metal-free, electrocatalyst, N_2_ fixation, Faradaic efficiency

## Abstract

The ever-growing requirement of ammonia for industry and energy supply motivates people to find clean and cost-effective alternatives to overcome the shortcomings of the century-old Haber-Bosch process. Electrocatalytic N_2_ reduction (NRR) is considered a prospective way for ammonia production at ambient conditions. Recently, two-dimensional (2D) metal-free materials are emerging as highly efficient and robust electrocatalysts for NRR owing to their advanced features: highly exposed surface, abundant active sites, tunable electronic states, and long-term stability compared to metal-based and bulk catalysts. In this minireview, we briefly summarize the latest edge reports on 2D metal-free materials catalyzed NRR. Also, we discuss the challenges and perspectives on the design and synthesis of novel 2D metal-free catalysts.

## Introduction

As a fundamental chemical with fast-growing industrial demand due to increasing population and economy, ammonia (NH_3_) is not only an essential source of manmade fertilizer, but also a promising candidate of carbon-free carrier of hydrogen energy (Schloegl, 2003; Service, 2014). The most important way for producing ammonia is the fixation (reduction) of atmospherically abundant dinitrogen gas (N_2_). Besides the natural biological N_2_ fixation by nitrogenase enzymes, to date, industrial-scale NH_3_ production is still relying on traditional Haber-Bosch process with iron based catalysts at high temperatures (350–600°C) and pressures (150–350 atm), which annually requires about 1.4% of global energy consumption and a considerable CO_2_ emission (Kandemir et al., 2013; van der Ham et al., 2014). Therefore, searching for alternative ways for inexpensive, clean, and sustainable ammonia production is imperative. In the past years, studies on biomimetic catalysis, photocatalysis, and photo(electro)catalysis for N_2_ fixation under mild conditions have emerged (Li et al., 2015; Wang et al., 2017). Among them, electrocatalytic nitrogen reduction reaction (NRR) is considered a promising process for its cost-effective and environ-friendly advantages (Seh et al., 2017; Lv et al., 2018b; Hao et al., 2019).

Generally, for a heterogeneous electrocatalyst, there are two possible mechanisms for NRR: associative and dissociative (Suryanto et al., 2019). The dissociative mechanism is relatively hard to proceed because it requires a high energy barrier to break the N≡N triple bond. In associative mechanism, hydrogenation of nitrogen may occur through distal or alternating pathways (Nazemi et al., 2018). Both of the two pathways need the participation of protons. However, on most heterogeneous catalyst surfaces, protons and electrons in aqueous solution prefer to undergo hydrogen evolution reaction (HER) rather than NRR, leading to low selectivity and unsatisfactory Faradaic efficiencies (FEs) (Singh et al., 2017). So far, a majority of reported NRR catalysts are based on noble/transition/p-block metals (Li et al., 2019; Wang et al., 2019). The scarcity of noble metals impedes their widespread application (Bao et al., 2017). For transition metals, the NH_3_ yield and FE are severely limited by the poor contact of N_2_ and catalyst surface as well as high HER competitiveness due to the easy formation of metal-H bonds (Su et al., 2012; Zheng et al., 2015; Guo et al., 2018).

In the past decade, metal-free electrocatalysts have attracted substantial attention in the field of energy conversion (Liu and Dai, 2016; Feng et al., 2017; Xie et al., 2018; Qin et al., 2019). Notably, metal-free catalysts also have huge potential for catalyzing NRR owing to their beneficial properties including good conductivity, controllable porosity, and intrinsic activity for N_2_ reduction (Zhao et al., 2019). Some metal-free catalysts can deliver favorable NRR selectivity due to the synergistic interactions derived by adjustable heteroatom doping. Moreover, metal-free catalyst could have good durability by avoiding the inherent corrosion susceptibility of metal-based catalysts in acidic/basic media (Chu et al., 2019). In the family of metal-free catalysts, two-dimensional (2D) materials (graphene, g-C_3_N_4_, boron sheet, black phosphorus, etc.) have been a research hotspot for the unique features absent from their bulk counterparts: high lateral-area-to-thickness ratio, tunable electronic states, and rich surface defects with active bonding sites. The above advances of 2D metal-free materials are greatly beneficial for constructing electrocatalytically active catalysts. In this minireview, we briefly summarize the recent progresses and challenges in carbon/boron/phosphorus based 2D metal-free electrocatalysts for NRR.

## Carbon-Based Catalysts

Although graphene has been widely explored with its intrinsic advantages such as high conductivity, pristine graphene, or chemically reduced graphene oxide (RGO), it is considered to have less catalytic activity. Doping with nonmetallic heteroatoms is an encouraging way for enhancing the catalytic performance of graphene. In recent years, there have been reports of nitrogen doped porous carbons/carbon nanospikes/boron-rich covalent organic frameworks (COFs) with superior catalytic activities for NRR (Liu et al., 2018b, 2019; Mukherjee et al., 2018; Song et al., 2018). The enhanced catalytic performance of doped carbon may be ascribed to the strong electronegativity of nitrogen dopants which positively charges the adjacent carbon atoms (Hu and Dai, 2017).

**Figure 1:**
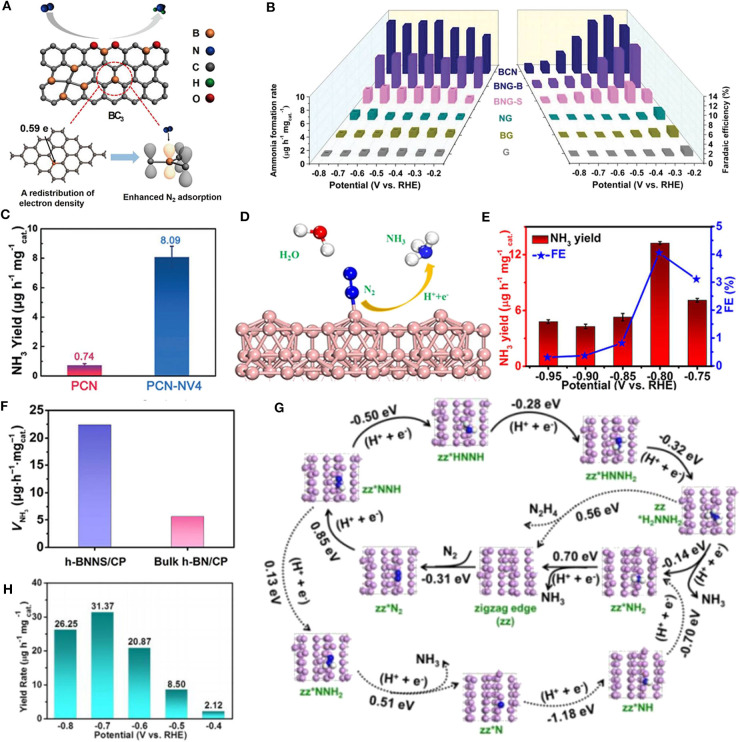
**(A)** Schematic illustration of boron doped graphene catalyzed NRR. BC_3_ structure provided the lowest energy barrier for N_2_ fixation reaction. Reproduced with permission from Yu et al. (2018). Copyright 2018 Cell. **(B)** NH_3_ yields and Faradaic efficiencies of relative catalysts. Reproduced with permission from Chen et al. (2019a). Copyright 2019 Wiley-VCH Verlag GmbH & Co. KGaA. **(C)** NH_3_ yields of carbon nitride with and without nitrogen vacancies at −0.2 V vs. RHE. Reproduced with permission from Lv et al. (2018a). Copyright 2018 Wiley-VCH Verlag GmbH & Co. KGaA. **(D)** Schematic illustration of boron nanosheet (BNS) catalyzed NRR. Compared to bare BNS, the B atoms of both oxidized and H-deactivated BNS have better activity for catalyzing NRR. **(E)** NH_3_ production rates and Faradaic efficiencies for BNS/CP at various potentials. Both **(D,E)** are reproduced with permission from Zhang et al. (2019b). Copyright 2019 American Chemical Society. **(F)** NH_3_ yields of h-BNNS/CP and bulk h-BN/CP at −0.75 V vs. RHE after 2 h electrolysis. Reproduced with permission from Zhang et al. (2019c). Copyright 2019 Springer. **(G)** Supposed NRR mechanisms at the zigzag edge of FL-BP NSs (solid line: low energy route, dotted line: unfavorable route). **(H)** NH_3_ yields of FL-BP NSs/CF at corresponding potentials. Both **(G,H)** are reproduced with permission from Zhang et al. (2019a). Copyright 2019 Wiley-VCH Verlag GmbH & Co. KGaA.

Unlike nitrogen, boron is less electronegative than carbon (2.04 vs. 2.55) (Yang et al., 2011). When forming a B-C bond, the doped B atom is positively charged with an empty orbital which can bind with the lone pair electrons in N_2_. Moreover, B can act as a Lewis acid site, which is beneficial to the adsorption of weak Lewis base N_2_ and repulsion of Lewis acid H^+^ in acidic media. Yu et al. introduced B into graphene framework by heating H_3_BO_3_ and graphene oxide in H_2_-Ar mixed atmosphere (Yu et al., 2018) (Figure 1A). The B doping did not change the typical planar structure of graphene, confirmed by transmission electron microscopy (TEM) images. X-ray photoelectron spectroscopy (XPS) showed the electron redistribution in graphene sheet by different B doping structures at the edge or defect sites. Temperature-programmed desorption (TPD) revealed that B atom could afford chemical adsorption sites for N_2_. Density functional theory (DFT) calculations indicated that in the several types of boron-doped carbon structures, B atom in BC_3_ is more positively charged. And BC_3_ structure provided the lowest energy barrier for N_2_ fixation reaction. The resulted catalyst presents a superior NH_3_ evolution rate of 9.8 μg h^−1^ cm^−2^ and excellent FE of 10.8% at −0.5 V vs. RHE, five and 10 times of those of undoped graphene, respectively.

It's worth noting that multiple dopants may work synergistically to improve catalytic performance. For instance, a metal-free electrocatalyst of boron/nitrogen co-doped carbon (BCN) is reported (Chen et al., 2019a) (Figure 1B). At first, B, N co-doped graphene was synthesized by annealing graphene oxide and boric acid in NH_3_ gas (BNG-B) or in Ar gas (BNG-S). In BNG-B, B-N pairs were formed through the reaction of boric acid and ammonia. The authors further prepared defect enriched BCN by pyrolysis of boric acid, urea, and PEG-2000 in Argon gas. DFT calculations and electrochemistry performance revealed that the co-doping of N and B was favorable for NRR and inactive for HER, which significantly promoted FE. In particular, the B-N pairs could act as active triggers for nitrogen reduction, and the adjacent edge carbon atoms provided the reactive sites. Thus, the doping of B-N pairs plays a key role in activating NRR as well as inhibiting the unfavorable HER process. As a result, the BCN catalyst delivered an excellent NH_3_ formation rate (7.75 μg h^−1^ mg^−1^_cat._) and Faradaic efficiency (13.79%) at −0.3 V vs. RHE, even outperforming most of the metal-based electrocatalysts.

Recently, Asiri, Sun, and their coworkers constructed oxygen doped porous carbon nanosheet (O-CNS) via a simple annealing process of sodium citrate in Ar (Chen et al., 2019b). The as-prepared catalyst delivered sheet-like structure confirmed by TEM images. The energy dispersive X-ray (EDX) elemental mapping and XPS suggested that O has been uniformly doped into carbon nanosheet. To evaluate the catalytic activity, NRR analysis was tested in 0.1 M HCl solution with O-CNS loading amount of 0.1 mg cm^−2^ on carbon paper. The as-obtained catalyst reached a high NH_3_ production of 18.03 μg h^−1^ mg^−1^_cat._ at −0.55 V and promising FE of 10.3% at −0.45 V with good durability. The same group also incorporated sulfur into graphene to form an efficient and stable electrocatalyst (S-G) for NRR (Xia et al., 2019). S-G catalyst was synthesized through a heat treatment process of graphene oxide with benzyl disulfide. TEM images showed that compared to undoped graphene, S-G presented the same nanosheet morphology. Raman spectra suggested that S-G was more disordered in structure than pristine graphene. DFT calculation demonstrated that by the substitution of sulfur, the neighboring carbon atoms could perform as active sites for N_2_ fixation. Consequently, this catalyst supported on carbon paper delivered outstanding electrochemical activity and stability (NH_3_ yield of 27.3 μg h^−1^ mg^−1^_cat._ at −0.6 V, FE of 11.5% at −0.5 V).

Graphitic carbon nitride (g-C_3_N_4_), as a metal-free catalyst with 2D layered structure, has been substantially studied for its promising application in photo/electrocatalytic reactions of energy conversion (Wang et al., 2012; Cao et al., 2015). It has been considered that vacancies on 2D catalyst materials may play the crucial role of providing active sites for surface reactions (Xie et al., 2020). Lv and Qian et al. proposed polymeric carbon nitride (PCN) with nitrogen vacancy (NV) defects (Lv et al., 2018a) (Figure 1C). The introduction of NVs can be easily controlled by a re-calcination process in Ar atmosphere. XRD and Fourier-transform infrared (FTIR) spectroscopy showed that the NVs destroyed long-range periodicity in PCN while inherited the short-range structure such as s-triazine ring and aromatic C-N heterocycles. Electron paramagnetic resonance (EPR) spectra further illustrated that the NVs could change the electron distribution on the nearby carbon atoms. Theoretical calculations reveal that N_2_ molecule could form a dinuclear end-on bound structure with NVs when adsorbing on PCN sheet, during which the N≡N bond of adsorbed N_2_ is strongly activated and stretches longer. In NRR test, the PCN with NVs defect engineering presented surprising electrocatalytic performance. Compared to bare PCN, PCN with NVs promoted NH_3_ yield for over 10-fold (8.09 μg h^−1^ mg^−1^_cat._), and a superior Faradaic efficiency of 11.59% was achieved at −0.2 V vs. RHE.

Very recently, Chu et al. incorporated sulfur dopants to occupy NVs of g-C_3_N_4_ by annealing NV-C_3_N_4_ in sulfur vapor (Chu et al., 2020). The as-prepared S-NV-C_3_N_4_ showed wrinkled sheet-like structure in TEM images, similar to NV-C_3_N_4_ precursor. XPS analysis proposed that rather than with nitrogen atoms, the doped sulfur preferred to interact with carbon atoms. In contrast to S-NV-C_3_N_4_, the doping of S significantly enhanced the NRR performance: a NH_3_ yield of 32.7 μg h^−1^ mg^−1^_cat._ and an FE of 14.1% at −0.4 V vs. RHE in 0.5 M LiClO_4_ as well as good durability for at least 20 h. In order to examine the underlying mechanism of activity promotion by S doping, DFT calculations were performed. It shows that S dopant played the role of stabilizing ^*^N_2_H and destabilizing ^*^NH_2_ to break the scaling relation of them. Moreover, the high Gibbs free energy for water dissociation and low free energy for H^*^ adsorption of S-NV-C_3_N_4_ suppressed undesired HER process, leading to a high Faradaic efficiency for N_2_-to-NH_3_ conversion.

## Boron-Based Catalysts

Boron carbide (B_4_C) has attracted much research attention as electrodes and catalyst backbones due to its good electronic conductivity and chemical stability (Mu et al., 2016; Song et al., 2017). In 2018, Qiu et al. demonstrated that B_4_C nanosheet could act as a promising metal-free electrocatalyst for N_2_ fixation both in acidic and neutral media (Qiu et al., 2018). The B_4_C nanosheet was simply prepared by ultrasonic exfoliating of commercial bulk B_4_C in ethanol. Few-layered B_4_C nanosheets can be seen from TEM images of samples after exfoliation. The catalytic mechanism is explored by DFT calculation; the rate-limiting step is considered as ^*^NH_2_-^*^NH_2_→^*^NH_2_-^*^NH_3_ reaction. NRR test is conducted to evaluate the electrochemical performance of as-prepared catalyst. In 0.1 M HCl, the B_4_C nanosheet exhibits superior NH_3_ yield of 26.57 μg h^−1^ mg^−1^_cat._ with a high Faradaic efficiency of 15.95% at −0.75 V. Moreover, this catalyst also behaves impressively in neutral media. In 0.1 M Na_2_SO_4_, the NH_3_ yield reaches 14.70 μg h^−1^ mg^−1^_cat._, and FE achieves 9.24%.

Boron has been considered to have unique bonding features due to its electron deficiency nature (Légaré et al., 2018; Liu et al., 2018a). It's noteworthy that a 2D boron sheet is reported to possess metallic character with high conductivity and good stability (Xu et al., 2015). Recently, Zhang and Wu et al. proposed boron nanosheet (BNS) as an elemental 2D electrocatalyst for efficient N_2_-to-NH_3_ reduction (Zhang et al., 2019b) (Figure 1D). The BNS was obtained by ultrasonic exfoliation of commercial bulk B powders in isopropyl alcohol (IPA) and supported on carbon paper (CP). Atomic force microscopy (AFM) images and height profile reveal the average thickness of BNS to be ~3.0 nm. N_2_ adsorption–desorption isotherms showed that BNS has remarkably enhanced surface area than that of bulk boron. In 0.1 M Na_2_SO_4_ solution, BNS/CP achieves inspiring NH_3_ yield of 13.22 μg h^−1^ mg^−1^_cat._ and Faradaic efficiency of 4.04% at −0.80 V vs. RHE as well as good durability (Figure 1E). The excellent electrocatalytic performance of BNS may come from the abundant exposing active sites in its 2D structure and better conductivity for electron transfer (confirmed by electrochemical impedance spectroscopy analysis) than bulk B/CP. Additionally, DFT calculations demonstrated that compared to clean BNS, the B atoms of both oxidized and H-deactivated BNS have better activity for catalyzing NRR, and the desorption of the second NH_3_ molecule determines the reaction rate.

In another independent report, Fan et al. proposed a liquid ultrasonic exfoliation method to efficiently synthesize single- and few-layer boron nanosheets from bulk boron (Fan et al., 2019). They found six organic solvents from 21 candidates suitable for exfoliation and dispersion boron, in which benzyl benzoate performs best. XPS showed that in boron nanosheets, the B-O peak was reduced compared to that in bulk boron, implying the dislodgment of oxygen-containing groups by exfoliation. Crystalline features analysis confirmed the β-rhombohedral B structure in as-obtained boron nanosheets. The average layer number of boron flakes was estimated to be about 13 layers, which corresponds to ca. 7.5 nm in thickness, denoted by AFM. In NRR test in 0.1 M HCl, the exfoliated boron nanosheets present a NH_3_ yield of 3.12 μg h^−1^ mg^−1^_cat._ and an FE of 4.84 % at −0.14 V, about a 2-fold enhancement to those of bulk boron. Theoretical calculations demonstrated that the icosahedron borons in 2D nanosheets could act as reactive sites and strongly adsorb N_2_ which benefits to the initial activation.

Although 2D hexagonal boron nitride (h-BN) has a wide band gap, ultrathin h-BN layers can narrow the band gap by introducing defects and improve conductivity by electron tunneling, thus deliver potential capability for catalyzing NRR. Zhang and Du et al. found that h-BN nanosheet (h-BNNS) exfoliated from bulk h-BN powders in ethanol could act as an efficient electrocatalyst for N_2_-to-NH_3_ reduction (Zhang et al., 2019c). AFM images showed that the average thickness of h-BNNS is about 1.3 nm. In NRR experiment, h-BNNS was supported on carbon paper with a loading amount of 0.1 mg cm^−2^, and tested in 0.1 M HCl solution. The catalyst achieved an encouraging NH_3_ yield of 22.4 μg h^−1^ mg^−1^_cat._ and a Faradic efficiency of 4.7% at −0.75 V vs. RHE with good stability in 24 h electrolysis (Figure 1F). DFT calculations further reveal that the energy barrier for NRR can be remarkably reduced through activating inert N_2_ molecule by the unsaturated boron at the edge site.

## Phosphorus-Based Catalysts

Phosphorus is an earth-abundant element widely used as dopants in catalysts. Briefly, elemental P exists in the following allotropes: white phosphorus (WP), red phosphorus (RP), violet phosphorus (VP), and black phosphorus (BP). Among them, few-layered black phosphorus (BP) has attracted numerous research interests as a star candidate in the family of 2D structured materials. BP has been applied in field-effect transistors, gas sensors, catalysis, and energy storage due to its high hole mobility, tunable band gaps, and unique chemical features (Pang et al., 2018; Wu et al., 2018; Xing et al., 2019; Shen et al., 2020). Notably, BP has a valence electron structure (3s^2^ 3p^3^) similar to that of nitrogen (2s^2^ 2p^3^). Hence, holding the concept of “like dissolves like.” Zhang et al. exfoliated orthorhombic black phosphorus into few-layer nanosheets (FL-BP NSs), which are used to catalyze NRR (Zhang et al., 2019a). AFM analysis showed that FL-BP NSs have a thickness of ~4.1 nm, corresponding to five to seven layers. The NRR activity test was performed in 0.01 M HCl. The FL-BP NSs exhibit dramatically high NH_3_ yield of 31.37 μg h^−1^ mg^−1^_cat._ at −0.7 V with FE of 5.07% at −0.6 V (Figure 1H). Moreover, the active sites for N_2_ activation are suggested to be the zigzag and diff-zigzag edges of the FL-BP NSs by DFT calculations (Figure 1G).

## Conclusion

In this minireview, the latest approaches in two-dimensional (2D) metal-free electrocatalysts for NRR are briefly summarized and discussed. Aiming to replace the Haber-Bosch process, research on electrochemical NRR has been one of the hottest points in catalysis and lots of inspiring works have been reported. However, the existing catalysts are still at a considerable distance from satisfactory NH_3_ yield and Faradaic efficiency for industrial use. Given the complicated reaction pathways of NRR accompanied by the competition of HER, the real active sites for N_2_ activation remains unclear and the exact catalytic mechanism need to be better understood. More profound insights on structure–function relationship should be obtained by further combination of experimental research and theoretical calculation. For the goal of improving NRR as well as suppressing HER, several strategies should be adopted. First, the elemental composition of current metal-free catalysts is still limited (B, C, N, P, S). The element choice for catalysts and dopants needs to be enlarged. Second, considering the advantages of 2D architecture, methods including defect engineering, doping control, adjusting surface states, and tailoring electronic structures should be carried out to optimize the catalytic performance. Third, to accurately evaluate the catalytic performance, quantitative detection of produced ammonia is of vital importance for data reliability. However, it's hard for traditional detection methods (indophenol blue, Nessler's reagent, etc.) to avoid N species contamination during the test, especially for N containing catalysts. Thus, the involvement of confirmation experiment using ^15^N isotope tracer is quite essential for the test proposal. At last, we anticipate that on the basis of developing highly active electrocatalysts, more attention should be put into the issues on the scalable and reproducible preparation of catalyst and fast fabrication of electrodes for the practical application of electrochemical N_2_ fixation in the future.

## Author Contributions

BT supervised the project. XX and BT mainly wrote the paper. BL and SL co-wrote the paper. All authors discussed the results and commented on the manuscript and agree to be accountable for the content of the work.

## Conflict of Interest

The authors declare that the research was conducted in the absence of any commercial or financial relationships that could be construed as a potential conflict of interest.
